# Identification of SSR Markers Correlated with Growth Traits in Swimming Crab (*Portunus trituberculatus*)

**DOI:** 10.3390/ijms27167169

**Published:** 2026-08-11

**Authors:** Baohua Duan, Yifan Shen, Boliang Yang, Yang Xu, Tongxu Kang, Shumei Mu, Xianjiang Kang

**Affiliations:** 1 College of Life Sciences, Hebei University, Baoding 071000, China; 2025210010@xyafu.edu.cn (B.D.); 15732285932@163.com (Y.S.); 15525865849@163.com (B.Y.); 18273912983@163.com (Y.X.); 13180587250@163.com (T.K.); shumeimu@126.com (S.M.); 2 College of Fisheries, Xinyang Agriculture and Forestry University, Xinyang 464000, China

**Keywords:** *Portunus trituberculatus*, SSR markers, growth traits, marker-assisted selection (MAS)

## Abstract

The swimming crab, *Portunus trituberculatus*, holds significant economic value in aquaculture, and marker-assisted selection (MAS) offers a powerful strategy to accelerate genetic improvement of growth-related traits. In this study, we performed an association analysis between 40 SSR markers and growth traits in 244 *P. trituberculatus* individuals. High genetic diversity was observed, with mean Shannon’s diversity index (*SI*) = 1.970, expected heterozygosity (*He*) = 0.773, and polymorphism information content (*PIC*) = 0.747. Linkage disequilibrium (LD) analysis revealed that all 485 marker pairs were in linkage equilibrium (*r*^2^ < 0.33), validating the suitability of these markers for association mapping. A total of eight SSR markers were identified as significantly associated with growth traits (*p* < 0.05, FDR < 0.05). Notably, marker PrMa04 was associated with six traits (FCW, CW, CL, MLC, BH, BW), suggesting pleiotropic effects. Marker TRAN20 explained the highest phenotypic variance (PVE = 17.06%) for CL. Sequence analysis revealed that PrMa01, PrMa05, and ZL06 are located near genes encoding zinc finger protein, receptor-type tyrosine-protein phosphatase, and mucin-2, respectively, which are known to be involved in growth and development. The eight candidate markers identified in the discovery population were not replicated in an independent cohort after FDR correction, suggesting that their effects may be population-specific. These findings provide valuable SSR markers and genetic insights for MAS programs in *P. trituberculatus*, while also highlighting the critical importance of cross-population validation before routine application.

## 1. Introduction

The swimming crab, *Portunus trituberculatus*, is a large portunid species widely distributed along the coastal waters of East Asia, particularly in China, Japan, and Korea [1]. Due to its rapid growth rate, high nutritional value, and strong market demand, it has become one of the most economically important marine aquaculture species in China, with annual production exceeding 450,000 tons in recent years [2]. Its culture industry has expanded rapidly, driven by the development of pond farming, polyculture systems, and enhanced juvenile production technologies. However, the intensive farming model has also brought about a series of challenges, including germplasm degradation, reduced disease resistance, and increasing environmental stress, which collectively constrain the sustainable development of the industry [3,4]. To address these issues, selective breeding programs aimed at improving growth performance and disease tolerance have been initiated [5,6]. Traditional phenotype-based selection, while effective to some extent, is often time consuming, labor intensive, and inefficient, especially for quantitative traits such as body weight and size, which are influenced by polygenic effects and environmental factors [7,8]. Therefore, the application of molecular-marker-assisted technologies has become an urgent necessity to accelerate genetic improvement in this species.

In recent decades, the rapid advancement of high-throughput sequencing and genotyping technologies has greatly facilitated the identification of molecular markers associated with economically important traits in aquatic animals. Among various types of molecular markers, simple sequence repeats (SSRs), also known as microsatellites, have been widely adopted due to their abundance, high polymorphism, co-dominant inheritance, reproducibility, and relatively low genotyping cost [9,10]. These attributes make SSRs particularly suitable for applications in population genetics, linkage mapping, and marker-assisted selection (MAS). MAS enables indirect selection for target traits based on linked markers, thereby increasing selection accuracy, shortening breeding cycles, and enhancing genetic gain, especially for traits that are difficult to measure, exhibit low heritability, or are expressed late in development [11,12]. To date, association analyses using SSR markers have been successfully conducted in a wide range of aquaculture species, leading to the identification of numerous markers significantly linked to growth. Notable examples include mandarin fish (*Siniperca chuatsi*) [13], silver carp (*Hypophthalmichthys molitrix*) [14], giant river prawn (*Macrobrachium rosenbergii*) [15], and mud crab (*Scylla paramamosain*) [16]. In finfish, MAS has been successfully applied to improve resistance to infectious pancreatic necrosis virus (IPNV) in Atlantic salmon (*Salmo salar*) [17] and to identify sex-linked markers for sex-controlled breeding in blue tilapia (*Oreochromis aureus*) [18]. These findings demonstrate the feasibility of SSR-based MAS in aquatic genetic improvement.

Despite the increasing availability of molecular markers and the growing body of association studies in other aquatic species, the application of SSR markers for MAS in *P. trituberculatus* remains relatively limited and fragmented. Early genetic studies on this species primarily focused on population genetic diversity and phylogeography using a small number of markers [19,20,21,22]. To date, several research groups have attempted to identify growth-related markers in *P. trituberculatus*. For instance, Liu et al. (2012) [23] screened a set of SSR markers for their association with growth-related traits in a cultured population, with Pot08 and Pot42 showing a significant impact on body weight and carapace length. However, these earlier investigations were mostly exploratory in nature, typically involving moderate sample sizes and relatively limited marker panels. In addition, comprehensive linkage disequilibrium (LD) analyses and simultaneous assessments of multiple growth-related traits have seldom been conducted in this species. As a result, the number of growth-associated SSR markers that have been validated for practical application remains limited. Therefore, further identification and validation of growth-associated SSR markers through extensive association studies are required to facilitate MAS in *P. trituberculatus*.

Here, a systematic association analysis was performed between 40 polymorphic SSR markers and seven key growth traits of *P. trituberculatus*. This study aimed to identify growth-associated SSR markers and investigate their potential biological functions. The results are expected to provide valuable genetic markers and foundational data for future MAS applications in this species.

## 2. Results

### 2.1. Phenotypic Variation and Correlation of Growth Traits

Descriptive statistics for the seven growth traits are presented in Table 1. Body weight (BW) exhibited the highest coefficient of variation (CV = 21.89%), suggesting substantial phenotypic plasticity and great potential for genetic improvement; claw-related traits (FLC, CV = 13.64%; MLC, CV = 13.93%) also showed relatively high variability, whereas carapace-related traits displayed lower CV values (8.02–8.60%), reflecting stable morphological characteristics within this population. Pearson correlation analysis revealed distinct association patterns among traits (Table 2). Strong and highly significant positive correlations (*p* < 0.01) were observed among FCW, CW, CL, BH, and BW, with coefficients ranging from 0.711 to 0.919. Notably, the exceptionally high correlation between FCW and BW (r = 0.919) suggests that full carapace width could serve as an effective indirect selection criterion for body weight. In contrast, FLC showed only weak or non-significant correlations with most other traits, except for modest correlations with CL (r = 0.158, *p* < 0.05) and MLC (r = 0.202, *p* < 0.01), indicating that claw length is largely under independent genetic control relative to overall body size. MLC exhibited moderate to strong correlations with body-size traits (r = 0.481–0.821), suggesting it is more closely linked to general body growth. These phenotypic associations provide a solid foundation for subsequent marker–trait association analyses aimed at dissecting the genetic architecture underlying growth in *P. trituberculatus*.

### 2.2. Genetic Diversity of SSR Markers

The genetic diversity parameters for the 40 SSR loci are summarized in Table 3. A total of 719 alleles were detected, with *Na* per locus ranging from 6 to 51 and a mean of 17.98. The mean *Ae* was 6.75 (range: 1.85–25.24). The *Ho* and *He* values averaged 0.684 and 0.773, respectively. The *PIC* values ranged from 0.438 (TRAN1) to 0.959 (PrMa05), with an average of 0.747, indicating that 38 of the 40 loci were highly polymorphic (*PIC* > 0.5). Significant deviations from *HWE* were observed at 33 loci (*p* < 0.01). Null alleles were detected in 37 loci (*Fna* > 0), which likely contributed to the *HWE* deviations.

Among the 40 SSR loci, six markers (DX09, PrMa03, PrMa05, TRAN15, TRAN19, and TRAN20) exhibited extremely high allelic richness (Na = 28–51) with a substantial proportion of rare alleles (minor allele frequency < 5%). In LD analysis using TASSEL, such markers with excessively rare alleles are automatically filtered out because they cannot provide reliable *r^2^* estimates due to inflated variance and unstable *p*-values. Consequently, these six markers were absent from the LD output, and only the remaining 34 markers yielded valid pairwise LD estimates. The rationale for this automatic exclusion is well-recognized in population genetics: markers with very low minor allele frequencies can produce spurious non-random associations that do not reflect true physical linkage.

### 2.3. Linkage Disequilibrium Analysis

**Figure 1:**
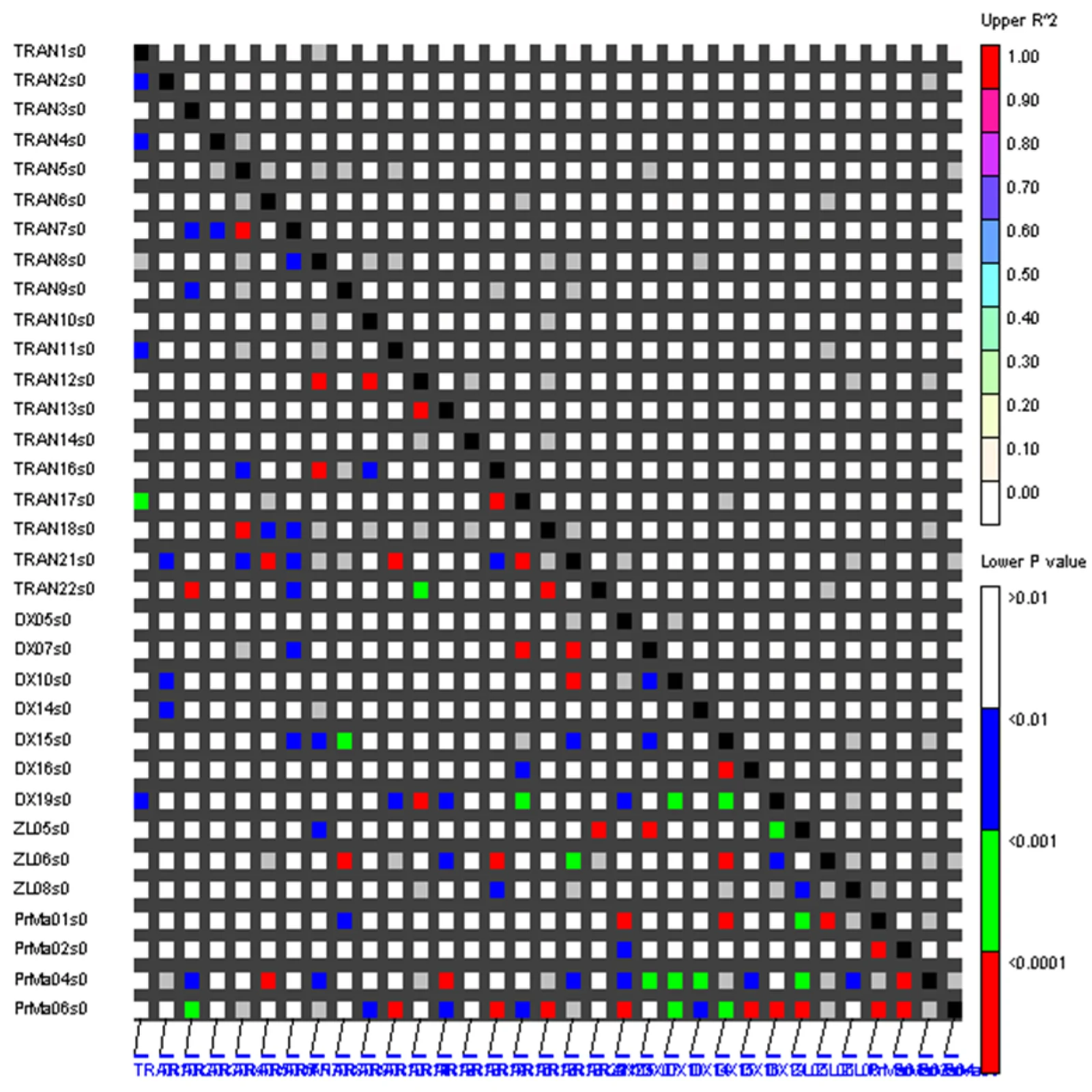
Linkage disequilibrium (LD) patterns among 244 individuals using 40 SSR markers. The upper triangle shows pairwise *r*^2^ values, and the lower triangle displays their corresponding significance levels. Color intensity represents *r*^2^ values (0.00–1.00), with significance thresholds indicated as *p* < 0.01 (blue), *p* < 0.001 (green), and *p* < 0.0001 (red).

The results of linkage disequilibrium are shown in Figure 1. All 40 SSR markers were used as input for the LD analysis in TASSEL. However, as described in Section 2.2, six markers with extremely high allelic richness and an excess of rare alleles (DX09, PrMa03, PrMa05, TRAN15, TRAN19, and TRAN20) were automatically filtered out by the software because reliable *r^2^* estimates could not be obtained due to inflated variance from low-frequency alleles. Consequently, only the remaining 34 markers yielded valid pairwise LD estimates. A total of 485 pairwise combinations were successfully generated from these markers, with *r^2^* ranging from 0.00258054 to 0.01078554 (mean = 0.005126525 < 0.33) (Supplementary Table S1), indicating that all marker loci are in a state of linkage equilibrium. Among these 485 combinations, 19.18% (93 pairs), 7.84% (38 pairs), and 11.75% (57 pairs) of the marker pairs showed significant LD at the *p* < 0.01, *p* < 0.001, and *p* < 0.0001 levels, respectively. The fact that 485 pairs (rather than the theoretical 561 pairs from 34 markers) were successfully analyzed reflects the automatic removal by the software of certain marker pairs for which LD could not be reliably estimated due to very low allele frequencies or insufficient sample sizes; this is common in LD analysis and does not affect the overall conclusion. Notably, three of the six markers absent from the LD output (PrMa03, PrMa05, and TRAN20) were identified as significantly associated with growth traits in Table 4. This is not a contradiction: these markers were automatically filtered from LD estimation due to statistical constraints (excessively rare alleles compromising reliable *r^2^* estimation), but they remain valid for association analysis, which operates on different statistical principles (GLM with population structure correction and FDR adjustment). The two analyses are independent and serve different purposes.

### 2.4. Correlation Analysis

Following correction for population structure and multiple testing, the GLM analysis identified eight SSR markers significantly associated with six growth traits (FCW, CW, CL, MLC, BH, and BW), with no significant associations detected for FLC (Table 4). Among these, marker PrMa04 showed pleiotropic effects, being associated with all six traits (PVE = 5.27–9.51%). Markers ZL06 and PrMa01 were associated with four and three traits, respectively (PVE = 6.31–9.18%). Body weight, a key economic trait, was associated with six markers (PrMa04, ZL06, PrMa01, PrMa05, PrMa03, and TRAN12), underscoring its polygenic nature. Notably, marker TRAN20 explained the highest phenotypic variance for a single trait, accounting for 17.06% of the variation in CL. Moreover, sequence annotation suggested that PrMa01, PrMa05, and ZL06 are located near genes potentially involved in cellular regulation and growth, including zinc finger protein, receptor-type tyrosine-protein phosphatase, and mucin-2.

### 2.5. Validation of Significant Markers

To evaluate the reproducibility of the eight candidate markers, we genotyped an additional 100 individuals from an independent cohort and tested the same marker–trait associations. As summarized in Supplementary Table S2, none of the eight SSR markers showed significant associations with any of the seven growth traits in this validation population after FDR correction (FDR > 0.05 for all marker–trait combinations). This lack of replication suggests that the initial associations may be population-specific, potentially due to differences in linkage disequilibrium (LD) structure, genetic background, or genotype-by-environment (G × E) interactions between the two cohorts. Alternatively, some of the initial associations in the F_3_C population may represent false positives that were not fully eliminated by the FDR correction, underscoring the need for more stringent statistical thresholds or larger sample sizes in future association studies. These findings highlight the critical importance of validating candidate markers across multiple independent populations before their implementation in routine breeding programs. Notably, the post hoc power analysis indicated that with n = 100, α = 0.05 (FDR-corrected), and medium effect size (f^2^ = 0.15), the statistical power was approximately 0.80; however, for small effect sizes (f^2^ = 0.05–0.10), power dropped to 0.30–0.55, limiting the cohort’s ability to detect the moderate effects observed in the discovery population (PVE = 5.27–17.06%).

## 3. Discussion

In this study, we performed a systematic association analysis between 40 polymorphic SSR markers and seven growth traits in a selected breeding population (F_3_C) of *P. trituberculatus*. After stringent correction for population structure and multiple testing, eight SSR markers were identified as significantly associated with six growth traits (Table 4). Among these, PrMa04 exhibited pleiotropic effects, being associated with all six traits (PVE = 5.27–9.51%). This suggests that PrMa04 may either be in proximity to a gene or regulatory element with broad effects on body growth or in linkage with multiple nearby QTLs. While the underlying mechanism remains to be elucidated, the multi-trait association makes PrMa04 a promising candidate for further validation and potential application in MAS. Conversely, body weight—the primary economic trait—was associated with six different markers (PrMa04, ZL06, PrMa01, PrMa05, PrMa03, and TRAN12), underscoring its polygenic architecture. This complexity implies that selection for BW would benefit from a multi-marker approach rather than reliance on a single marker. Notably, TRAN20 explained the highest proportion of phenotypic variance for a single trait (17.06% for CL), indicating a relatively large effect on carapace length. Given the low LD observed across the marker panel, this marker may be in proximity to a QTL affecting CL, although fine-mapping or denser marker coverage would be required to confirm this. The substantial variation in PVE values among markers (5.27–17.06%) reflects the quantitative nature of growth traits and the varying effect sizes of individual loci, which is consistent with findings in other crustacean species [24,25,26]. However, when these eight markers were tested in an independent population, none of the associations remained significant after FDR correction (Supplementary Table S2). This lack of replication is not uncommon in association studies of complex quantitative traits, particularly in non-model species where LD decays rapidly, and QTL effects are often population-specific [27,28]. It is important to note that this failure does not indicate that the association method itself (GLM with population structure correction and FDR adjustment) was unsuitable. Rather, it reflects the inherent complexity of dissecting polygenic traits in genetically heterogeneous populations, where QTL effects are frequently context-dependent and may not transfer across populations with different genetic backgrounds, LD structures, or environmental conditions. This underscores the critical necessity of cross-population validation for candidate markers before they can be confidently applied in breeding programs.

The absence of significant associations with FLC, despite the identification of markers for all six other traits, warrants attention. This finding aligns with the weak phenotypic correlations observed between FLC and other growth traits (r = 0.074–0.202), further supporting the hypothesis that claw length is genetically independent from overall body growth [23,29]. This suggests that breeding programs aiming to improve claw-related traits would require separate marker discovery efforts and selection strategies. Sequence annotation revealed that PrMa01, PrMa05, and ZL06 are located near genes encoding zinc finger protein, receptor-type tyrosine-protein phosphatase, and mucin-2, respectively. Zinc finger proteins are transcription factors regulating cell proliferation, differentiation, and apoptosis; polymorphisms in such genes could influence growth by modulating muscle development or metabolic pathways [30,31,32]. Receptor-type tyrosine-protein phosphatases play critical roles in signal transduction, controlling cell growth, adhesion, and migration [33]. Mucin-2, a major component of intestinal mucus, is primarily associated with immune defense and gut health, but improved nutrient absorption efficiency could indirectly promote growth [34,35]. The remaining five associated markers (e.g., PrMa04, TRAN20) were not located in annotated gene regions, suggesting possible locations in intergenic regions or uncharacterized regulatory elements, which is not uncommon in non-model species with limited genomic resources.

The strong positive correlations among FCW, CW, CL, BH, and BW (r = 0.711–0.919) are consistent with previous reports in *P. trituberculatus* and other crustacean species [23,36,37], indicating that these traits are influenced by shared genetic or physiological pathways. The exceptionally high correlation between FCW and BW (r = 0.919) is of practical importance, as it suggests that FCW—a trait that is both heritable and easily measurable—could serve as a reliable indirect selection criterion for body weight [38]. This could simplify phenotyping in large-scale breeding programs, as FCW measurement is less sensitive to short-term environmental fluctuations (e.g., feeding status) than direct weighing. In contrast, the weak correlations of FLC and MLC with other traits indicate that claw dimensions are governed by largely independent genetic networks and would require separate selection strategies.

The genetic diversity parameters observed in this population (*PIC* = 0.747, *Na* = 17.98) indicate high levels of polymorphism and substantial allelic richness. These values are notably higher than those reported in several previous studies on wild or early-generation cultured populations of *P. trituberculatus* [19,20], suggesting that the F_3_C population has retained considerable genetic variability through controlled breeding practices—a favorable condition for ongoing selection. However, the high frequency of HWE deviations (33 of 40 loci) is likely attributable to a combination of factors. First, as a third-generation selected breeding population, the F_3_C population has undergone three generations of artificial selection for growth-related traits, which can disrupt HWE by favoring certain genotypes. Second, the population is maintained as a closed population with a limited number of founders, potentially leading to inbreeding. Third, the presence of null alleles (detected at 37 loci) contributes to heterozygote deficiencies. These factors are common in aquaculture breeding populations and do not invalidate the association analysis, particularly given the use of FDR correction and population structure covariates [39,40]. The high frequency of null alleles warrants caution in genotype calling, although the use of FDR correction and population structure covariates in our association analysis helps mitigate potential false positives. Continued genetic monitoring and careful mating design will be essential to manage inbreeding and maintain long-term genetic gain in this breeding program. In addition, null alleles were detected at 37 loci, a common occurrence in microsatellite studies of non-model species. While null alleles can contribute to heterozygote deficiencies and HWE deviations, their impact on the association results is mitigated by the use of FDR correction for multiple testing and population structure covariates in the GLM. Furthermore, none of the eight markers significantly associated with growth traits had high null allele frequencies (Fna > 0.2), suggesting that null alleles did not systematically drive the observed associations. To more rigorously assess the potential impact of null alleles on our findings, we performed a sensitivity analysis by excluding loci with Fna > 0.2 (TRAN21, PrMa02, PrMa06, and DX15) and re-running the GLM association analysis. This exclusion left 36 markers for analysis. The results showed that all eight markers originally identified as significantly associated with growth traits remained significant (*p* < 0.05, FDR < 0.05) after the exclusion of high-Fna loci (Supplementary Table S3). The PVE values for these markers remained largely unchanged (mean difference = 0.21 ± 0.14%), confirming that null alleles did not systematically drive the observed associations.

The failure to replicate the initial associations in the independent validation population warrants further consideration. Several non-mutually exclusive explanations may account for this discrepancy. First, the F_3_C and validation cohorts may differ in their extent of LD. If the eight SSR markers are not in close physical proximity to the causal variants, the LD between the marker and the QTL may break down across populations with different recombination histories or effective population sizes [41]. Second, genotype-by-environment (G × E) interactions could modulate the expression of growth-related QTLs, as the two cohorts were reared in different ponds, potentially leading to differential QTL effects [42]. Third, the validation population was smaller (n = 100) than the experimental population (n = 244), which reduced statistical power to detect small-to-moderate effect sizes. Post hoc power analysis indicated that with n = 100, the validation cohort was severely underpowered to detect small-to-medium effect sizes (f^2^ < 0.10), which characterized most of the associations observed in the discovery population (PVE = 5.27–9.51% for six of the eight markers). This explains why some marker–trait combinations showed nominal significance (*p* < 0.05) in the validation cohort, but none survived FDR correction: the cohort was underpowered to detect the moderate effects after multiple testing correction. Fourth, despite FDR correction, the initial associations may include a proportion of false positives—a known challenge in association studies with moderate sample sizes and marker densities. The fact that associations identified in the discovery population failed to replicate in an independent cohort, despite both populations originating from the same F3 generation and comparable rearing conditions, provides empirical evidence that even modest differences in population composition or micro-environmental conditions can substantially affect marker–trait associations [43]. This observation is consistent with findings in other aquaculture species, where QTLs detected in one population often fail to transfer to others due to population-specific LD structures, genotype-by-environment interactions, or differences in allele frequencies [44,45]. These results do not invalidate the association mapping approach; rather, they highlight the necessity of moving beyond single-population discovery toward multi-population validation as an integral component of marker development for MAS. Collectively, these findings emphasize that candidate markers identified in a single population should be interpreted with caution, and that rigorous validation across multiple, larger, and environmentally diverse populations is essential for the successful implementation of MAS in *P. trituberculatus* breeding programs.

The complete linkage equilibrium observed among all 485 marker pairs (all *r*^2^ << 0.33) confirms the absence of strong physical linkage within this marker set. This condition is essential for reliable association mapping, as high LD could otherwise generate spurious marker–trait associations [27]. The low LD observed here is consistent with findings in other crustacean species with relatively large effective population sizes [46,47] and further supports the robustness of our association results. The markers identified in this study can be readily integrated into a pragmatic MAS scheme for broodstock screening, with markers such as PrMa04 (pleiotropic) and TRAN20 (high PVE) offering immediate value. However, several limitations should be acknowledged. First, the sample size (n = 244) and marker density (40 SSRs) are moderate; larger cohorts and denser marker panels—such as SNP arrays—would facilitate more precise mapping of causal variants [48]. Second, the failure to replicate the initial associations in the independent validation population may partially reflect environmental non-comparability. While all individuals originated from the same F3 generation and were reared under standardized farm protocols, we lacked concrete daily environmental measurements (e.g., temperature, salinity, dissolved oxygen) for each pond. Thus, we cannot rule out the possibility that genotype-by-environment (G × E) interactions, or simply undetected micro-environmental differences between ponds, contributed to the lack of replication. This is a real and substantive limitation of the present study. Future validation studies should incorporate systematic environmental monitoring to explicitly account for G × E effects. Third, the associations are based on a single population (F_3_C) and require validation in independent populations before routine application. Fourth, the functional predictions for PrMa01, PrMa05, and ZL06 are based on sequence homology and require experimental verification through gene expression or functional studies. Finally, we acknowledge that molt stage was not standardized in this study. In crustaceans, morphometric measurements can vary with the time elapsed since the last molt. While all individuals were sampled at the same time from the same farm under standardized production conditions, the lack of molt stage standardization represents a potential source of environmental variance that could affect phenotypic measurements and genetic associations. Future studies should consider incorporating molt stage assessment into the phenotyping protocol.

Despite these limitations, the present study contributes to the ongoing breeding program of *P. trituberculatus* in several meaningful ways. First, the comprehensive genetic diversity parameters (*PIC* = 0.747, *Na* = 17.98) provide a baseline assessment of the F_3_C population’s genetic variability, confirming that it retains substantial allelic richness—a favorable condition for continued selection. Second, the LD characterization (all *r*^2^ < 0.33) confirms rapid LD decay in this species, providing critical guidance for the design of future association studies: denser marker panels (e.g., SNP arrays) will be required for effective genome-wide association and genomic selection. Third, the candidate gene annotations for PrMa01, PrMa05, and ZL06 offer biologically plausible targets for future functional studies, even though their immediate utility for MAS is not supported by the validation data. However, it must be acknowledged that the eight markers identified in this study are not immediately deployable for routine MAS in commercial breeding. The failed validation underscores that candidate markers identified in a single population should be interpreted with caution, and that rigorous validation across multiple, larger, and environmentally diverse populations is essential before any marker can be confidently integrated into breeding programs. This conclusion, while limiting the immediate applicability of our markers, provides a realistic and empirically grounded perspective on the challenges of implementing MAS in aquaculture species—a perspective that is often absent in the literature, where positive results are preferentially reported.

## 4. Materials and Methods

### 4.1. Experimental Population and Phenotyping

A total of 244 individuals from a third-generation selected breeding population (F_3_C) were sampled from a single pond at the National Swimming Crab Breeding Farm in Huanghua, Hebei Province, China (38°49′ N, 117°64′ E). Before tissue collection, individuals were placed on ice to reduce activity and facilitate handling. For each individual, seven growth-related traits were measured: body weight (BW), full carapace width (FCW), carapace width (CW), carapace length (CL), fixed length of the claw (FLC), meropodit length of the claw (MLC), and body height (BH), according to the previous study [36].

### 4.2. DNA Extraction and SSR Genotyping

Muscle tissue was dissected from the claws, and genomic DNA was extracted using the DNeasy Blood & Tissue Kit (Qiagen, Hilden, Germany). DNA quality and concentration were assessed using a NanoDrop 2000 spectrophotometer (Thermo Fisher Scientific Inc., Waltham, MA, USA). DNA integrity was checked on 1% agarose gels. From an initial 450 SSR primer pairs designed from transcriptome sequencing data, 40 polymorphic markers were selected through a two-step screening process. First, PCR amplification was performed using 7 DNA samples, and products were evaluated on 1% agarose gels to identify primers producing clear and reproducible bands. Second, the remaining products were subjected to 8% non-denaturing polyacrylamide gel electrophoresis followed by silver staining and gel imaging to assess polymorphism. Only markers that showed clear, reproducible banding patterns and detectable polymorphism in at least 4 of the 7 test samples were retained [20,22]. Primer pairs for each SSR locus were designed using Primer3 (http://primer3.sourceforge.net/releases.php) (accessed on 3 April 2021). The forward primer was labeled with the fluorescent dye, 6-carboxy-fluorescein (FAM) (General Biol, Chuzhou, China). PCR amplification was performed in a 10 μL reaction volume containing 5 μL of 2× Es Taq Master Mix (CWBIO, Beijing, China), 1 μL each of forward and reverse primers, 1 μL of DNA template, and 2 μL of RNase-free water. PCR conditions included an initial denaturation at 95 °C for 5 min, followed by 35 cycles of denaturation at 95 °C for 30 s, annealing at 60 °C for 30 s, and extension at 72 °C for 30 s, with a final extension at 72 °C for 10 min. PCR products were analyzed via capillary electrophoresis (CE) on an ABI-3730XL Genetic Analyzer (Applied Biosystems, Foster City, CA, USA) to obtain precise allele sizes and genotypes according to the previous study [21].

### 4.3. Data Analysis

#### 4.3.1. Genetic Diversity 

Genetic diversity parameters, including the number of alleles (*Na*), effective number of alleles (*Ae*), observed heterozygosity (*Ho*), expected heterozygosity (*He*), and Shannon’s information index (*SI*), were calculated using PopGene 1.32 [49]. Polymorphism information content (*PIC*) was computed using PICcalc software (version 0.6) [50]. Hardy–Weinberg equilibrium (*HWE*) and null allele frequencies (*Fna*) were assessed using GENEPOP [51].

#### 4.3.2. Correlation Analysis and LD Analysis

To minimize false-positive associations, the population structure (Q matrix) of the 244 individuals was estimated using STRUCTURE version 2.3.4 [52]. This Q matrix was then used as a covariate in a general linear model (GLM) implemented in TASSEL 2.1 [53] to test for associations between SSR markers and seven growth traits. In addition, the phenotypic variation explained (PVE) by each marker was also calculated. To correct for multiple testing, the Benjamini–Hochberg method was used to control the false discovery rate (FDR). Only marker–trait associations with *p* < 0.05 and FDR < 0.05 were considered statistically significant [54]. Phenotypic correlations among the seven growth traits were calculated using Pearson’s correlation coefficient in SPSS 22.0. Linkage Disequilibrium (LD) between all pairs of SSR markers was estimated using TASSEL 2.1 software, with the magnitude of LD expressed as *r*^2^ (squared correlation coefficients). A threshold of *r*^2^ > 0.33 was used to indicate tight linkage, following previous association studies [55]. This threshold was selected for two reasons. First, with a limited number of SSR markers (40 markers), a stringent threshold such as *r*^2^ > 0.8 is overly conservative and would fail to detect moderate LD that could still influence marker independence in association mapping. The *r*^2^ > 0.33 threshold has been empirically used in livestock association studies to flag markers requiring caution in interpreting independent effects [55]. Second, and more importantly, the purpose of LD analysis in this study was not to define “strong LD” per se, but rather to verify that the marker set as a whole is in linkage equilibrium, thereby validating its suitability for association analysis. As will be shown in the Results (Section 2.3), the observed *r*^2^ values were far below this threshold, confirming that the markers are in linkage equilibrium regardless of the specific threshold applied.

#### 4.3.3. Validation of Associated Markers in an Independent Population

To validate the SSR markers that were significantly associated with growth traits in the F_3_C population, an independent cohort of 100 individuals was sampled from the same F3 generation at the same farm but from different ponds. The commercial farm maintains standardized management protocols across all ponds, including target ranges for temperature, salinity, dissolved oxygen, feeding regime, and stocking density; however, routine monitoring records were not systematically archived for retrospective analysis. We acknowledge that specific daily environmental measurements for each pond were not available, and environmental comparability between cohorts is assumed based on farm-level management protocols rather than on empirical data. We have explicitly acknowledged this limitation in the Discussion. 

DNA extraction, PCR amplification, and SSR genotyping were performed following the same protocols as described in Section 4.2. The GLM, incorporating population structure (Q matrix) as a covariate and FDR correction for multiple testing, was applied to test for associations between the candidate markers and the seven growth traits in this validation population, using the same significance thresholds (*p* < 0.05 and FDR < 0.05) as in the discovery population. To ensure consistency in allele calling between the discovery and validation populations, the same genotyping protocol, PCR conditions, capillary electrophoresis platform (ABI-3730XL, Applied Biosystems, Foster City, CA, USA), internal size standard (GeneScan™ 500 LIZ^®^, Applied Biosystems, Waltham, MA, USA), and allele binning parameters in GeneMapper^®^ software (version 6.1) were used for both cohorts. Positive control DNA samples were included in each run to monitor consistency. 

Post hoc power analyses were conducted using G*Power software (version 3.1) to evaluate the statistical power of both the discovery (n = 244) and validation (n = 100) cohorts for detecting associations with effect sizes corresponding to the PVE values observed in this study. For the GLM with up to 7 predictors (6 growth traits + population structure covariate) and α = 0.05 (FDR-corrected), the power to detect medium effects (f^2^ = 0.15) was approximately 0.80 for n = 100 and >0.95 for n = 244. However, for small-to-medium effects (f^2^ = 0.05–0.10), power dropped to 0.30–0.55 for n = 100, indicating that the validation cohort was underpowered to detect the moderate effects that characterized most of the associations observed in the discovery population. No a priori power analysis was conducted, as sample sizes were determined by the available number of individuals from the F3 generation rather than prospectively.

#### 4.3.4. Functional Annotation of Candidate Markers

Functional annotation of the associated SSR markers was performed by extracting flanking sequences from the transcriptome assembly and subjecting them to BLASTx (BLAST, version 2.13.0) searches against the NCBI non-redundant (NR) protein database with an E-value threshold of 1 × 10^−5^. The best hit with the highest identity and lowest E-value was considered the putative functional annotation. As the markers were developed from transcriptome sequences rather than a genome assembly, the physical distance between the SSR motif and the annotated gene could not be precisely determined; the markers are located within or adjacent to the transcribed sequences showing homology to the candidate genes.

## 5. Conclusions

In conclusion, this study identified eight SSR markers significantly associated with growth traits in *P. trituberculatus*, including the pleiotropic marker PrMa04 and the high-effect marker TRAN20. The strong correlations among major body dimensions support the feasibility of indirect selection for body weight using easily measurable traits such as full carapace width. These candidate markers constitute a valuable genetic resource for marker-assisted selection in this species; however, the lack of replication in an independent population after FDR correction underscores the necessity of rigorous validation across multiple cohorts before these markers can be confidently implemented in commercial breeding programs.

## Figures and Tables

**Table 1:** Statistical data of growth traits of the *P. trituberculatus* population (F_3_C).

Trait	Min	Max	Mean ± SD	CV (%)
FCW (mm)	108.21	172.39	144.43 ± 11.58	8.02
CW (mm)	40.89	82.24	65.95 ± 5.67	8.60
CL (mm)	54.31	96.86	67.68 ± 5.61	8.29
FLC (mm)	54.45	104.77	73.18 ± 9.98	13.64
MLC (mm)	31.19	69.34	47.3 ± 6.59	13.93
BH (mm)	22.60	49.54	34.75 ± 2.84	8.17
BW (g)	69.12	267.14	152.43 ± 33.36	21.89

**Table 2:** Correlation analysis of growth traits of *P. trituberculatus* populations.

Trait	FCW	CW	CL	FLC	MLC	BH	BW
FCW	1						
CW	0.845 **	1					
CL	0.819 **	0.878 **	1				
FLC	0.082	0.119	0.158 *	1			
MLC	0.655 **	0.725 **	0.821 **	0.202 **	1		
BH	0.867 **	0.758 **	0.711 **	0.074	0.481 **	1	
BW	0.919 **	0.874 **	0.881 **	0.119	0.708 **	0.856 **	1

* *p* < 0.05; ** *p* < 0.01.

**Table 3:** Genetic diversity parameters of *P. trituberculatus* in the F_3_C population.

Locus	*Na*	*Ae*	*SI*	*Ho*	*He*	*PIC*	*Fna*	*HWE*
TRAN1	9	1.849	1.016	0.475	0.459	0.438	0.022	**
TRAN2	10	2.680	1.224	0.619	0.627	0.570	0.000	NS
TRAN3	10	1.933	1.142	0.443	0.483	0.465	0.022	NS
TRAN4	9	3.656	1.541	0.738	0.727	0.683	0.029	**
TRAN5	18	3.631	1.679	0.631	0.725	0.687	0.057	**
TRAN6	9	3.252	1.426	0.680	0.693	0.649	0.026	**
TRAN7	9	3.817	1.570	0.693	0.738	0.698	0.099	**
TRAN8	17	3.843	1.842	0.730	0.740	0.720	0.061	**
TRAN9	23	4.549	2.019	0.521	0.780	0.757	0.151	**
TRAN10	12	2.640	1.321	0.516	0.621	0.572	0.074	**
TRAN11	20	5.896	2.150	0.697	0.830	0.812	0.069	**
TRAN12	23	9.167	2.522	0.566	0.891	0.882	0.181	**
TRAN13	6	2.369	1.043	0.541	0.578	0.501	0.043	NS
TRAN14	9	3.169	1.385	0.652	0.684	0.638	0.078	**
TRAN15	26	10.447	2.686	0.857	0.904	0.898	0.036	**
TRAN16	15	2.458	1.354	0.590	0.593	0.558	0.034	**
TRAN17	17	7.143	2.202	0.635	0.860	0.845	0.117	**
TRAN18	15	5.608	1.983	0.820	0.822	0.801	0.020	**
TRAN19	33	11.714	2.855	0.779	0.915	0.909	0.065	**
TRAN20	28	17.823	3.018	0.844	0.944	0.941	0.053	**
TRAN21	28	7.427	2.414	0.492	0.865	0.854	0.203	**
TRAN22	10	4.805	1.708	0.725	0.792	0.762	0.031	NS
TRAN23	29	17.812	3.054	0.902	0.944	0.941	0.031	**
DX05	12	6.261	2.022	0.869	0.840	0.822	0.001	NS
DX07	6	3.054	1.297	0.613	0.673	0.614	0.052	**
DX09	36	18.319	3.126	0.803	0.945	0.943	0.077	**
DX10	11	3.900	1.596	0.725	0.744	0.706	0.040	**
DX14	10	2.322	1.159	0.557	0.569	0.525	0.020	**
DX15	15	8.364	2.283	0.521	0.880	0.869	0.212	**
DX16	10	3.790	1.476	0.676	0.736	0.691	0.037	NS
DX19	12	5.162	1.880	0.943	0.806	0.781	0.000	**
ZL05	11	2.846	1.316	0.471	0.649	0.593	0.106	**
ZL06	16	6.412	2.136	0.713	0.844	0.827	0.104	**
ZL08	17	5.506	2.027	0.861	0.818	0.800	0.006	**
PrMa01	18	5.510	2.052	0.926	0.819	0.798	0.000	NS
PrMa02	13	4.151	1.730	0.574	0.759	0.725	0.426	**
PrMa03	40	15.602	3.037	0.828	0.936	0.932	0.064	**
PrMa04	35	7.057	2.547	0.799	0.858	0.847	0.049	**
PrMa05	51	25.243	3.487	0.803	0.960	0.959	0.086	**
PrMa06	21	8.621	2.494	0.516	0.884	0.874	0.201	**
Mean	17.975	6.745	1.970	0.684	0.773	0.747	0.075	-

*Na*: number of alleles; *Ae*: number of effective alleles; *SI*: Shannon’s diversity index; *Ho*: observed heterozygosity; *He*: expected heterozygosity; *Fna*: null allele frequencies; *HWE*: Hardy–Weinberg equilibrium; ** *p* < 0.01; NS: no deviations from *HWE*.

**Table 4:** Association analysis between SSR markers and growth traits of *P. trituberculatus*.

Locus	Trait	PVE (%)	*p*	FDR
PrMa04	FCW	7.43	****	0.000104
	CW	6.98	****	0.000019
	CL	7.80	****	0.000001
	MLC	5.27	**	0.004316
	BH	9.51	****	0.000000
	BW	6.92	****	0.000001
ZL06	FCW	9.18	***	0.004048
	CW	8.81	****	0.000481
	BH	8.27	**	0.001939
	BW	6.80	**	0.005576
PrMa01	CW	6.31	***	0.004353
	BH	6.89	**	0.001486
	BW	6.93	***	0.000218
PrMa05	CW	7.01	***	0.005275
	BW	6.28	**	0.003795
PrMa03	BH	10.63	***	0.000125
	BW	8.11	**	0.001946
TRAN12	BH	7.22	**	0.009667
	BW	6.49	**	0.008000
TRAN20	CL	17.06	***	0.003703
DX19	BH	6.68	**	0.009806

PVE: phenotypic variation explained by each marker; FDR: false discovery rate; ** *p* < 0.01, *** *p* < 0.001, **** *p* < 0.0001.

## Data Availability

The original contributions presented in this study are included in the article and supplementary material. Further inquiries can be directed to the corresponding author.

## Supplementary Materials

The following supporting information can be downloaded at: https://www.mdpi.com/article/10.3390/ijms27167169/s1.
